# Supplementary material for: A fault-intrusion-tolerant system and deadline-aware algorithm for scheduling scientific workflow in the cloud
Source: PeerJ Comput Sci. 2021 Nov 2;7:e747. doi: 10.7717/peerj-cs.747 (PMC8576554; doi:10.7717/peerj-cs.747)
Supplement: Supplemental Information 6 [file peerj-cs-07-747-s006.zip › Myproject1.1/docs/org/workflowsim/clustering/balancing/methods/HorizontalImpactBalancing.html]

HorizontalImpactBalancing


JavaScript is disabled on your browser.


- Overview
- Package
- Class
- Use
- Tree
- Deprecated
- Index
- Help

- Prev Class
- Next Class

- Frames
- No Frames

- All Classes

- Summary:
- Nested |
- Field |
- Constr |
- Method

- Detail:
- Field |
- Constr |
- Method


org.workflowsim.clustering.balancing.methods

## Class HorizontalImpactBalancing

- java.lang.Object
- - org.workflowsim.clustering.balancing.methods.BalancingMethod
  - - org.workflowsim.clustering.balancing.methods.HorizontalImpactBalancing

- Direct Known Subclasses:
  :   HorizontalDistanceBalancing

  ---

    

  ```
  public class HorizontalImpactBalancing
  extends BalancingMethod
  ```

  HorizontalImpactBalancing is a method that merges tasks that have similar impact
  factors.

  Since:
  :   WorkflowSim Toolkit 1.0

- - ### Constructor Summary

    Constructors

    Constructor and Description

    `HorizontalImpactBalancing(java.util.Map levelMap,
    java.util.Map taskMap,
    int clusterNum)`

    Initialize a HorizontalImpactBalancing object
  - ### Method Summary

    Methods

    | Modifier and Type | Method and Description |
    | --- | --- |
    | `protected TaskSet` | `getCandidateTastSet(java.util.ArrayList<TaskSet> taskList, TaskSet checkSet, int clusters_size)` Gets the potential candidate taskSets to merge |
    | `void` | `process(java.util.ArrayList<TaskSet> taskList)` Sort taskSet based on their impact factors and then merge similar taskSet together |
    | `void` | `run()` The main function |

    - ### Methods inherited from class org.workflowsim.clustering.balancing.methods.BalancingMethod

      `addTaskSet2TaskSet, cleanTaskSetChecked, getClusterNum, getLevelMap, getTaskMap`
    - ### Methods inherited from class java.lang.Object

      `clone, equals, finalize, getClass, hashCode, notify, notifyAll, toString, wait, wait, wait`

- - ### Constructor Detail


    - #### HorizontalImpactBalancing

      ```
      public HorizontalImpactBalancing(java.util.Map levelMap,
                               java.util.Map taskMap,
                               int clusterNum)
      ```

      Initialize a HorizontalImpactBalancing object

      Parameters:
      :   `levelMap` - the level map
      :   `taskMap` - the task map
      :   `clusterNum` - the clusters.num
  - ### Method Detail


    - #### run

      ```
      public void run()
      ```

      The main function

      **Overrides:**
      :   `run` in class `BalancingMethod`


    - #### process

      ```
      public void process(java.util.ArrayList<TaskSet> taskList)
      ```

      Sort taskSet based on their impact factors and then merge similar taskSet together

      Parameters:
      :   `taskList` -


    - #### getCandidateTastSet

      ```
      protected TaskSet getCandidateTastSet(java.util.ArrayList<TaskSet> taskList,
                                TaskSet checkSet,
                                int clusters_size)
      ```

      Gets the potential candidate taskSets to merge

      Parameters:
      :   `taskList` -
      :   `checkSet` -

      Returns:


- Overview
- Package
- Class
- Use
- Tree
- Deprecated
- Index
- Help

- Prev Class
- Next Class

- Frames
- No Frames

- All Classes

- Summary:
- Nested |
- Field |
- Constr |
- Method

- Detail:
- Field |
- Constr |
- Method
